# Correspondence

**Published:** 1877-04

**Authors:** H. Gibbons

**Affiliations:** San Francisco


### Correspondence.

SAN FRANCISCO, March 15th, 1877.

EDITOR JOURNAL AND EXAMINER:

*Dear Doctor:*—In the March number of your excellent JOURNAL, is a reference to an article of mine in the *Pacific Medical and Surgical Journal* for January, on the use of tincture of iron in Diptheria, in which I am represented as saying that an adult patient should get from half an ounce to an ounce in twenty-four hours. By referring to the article you will find my language to be as follows: "From half an ounce to an ounce should be administered in twenty-four hours—the smaller quantity mentioned for a child of two years, and the quantity increased for older children." I am also quoted as advising as a substitute in case of the rejection of the tincture by the stomach, the tartrate of potassa. My proposed substitutes was the *citrate or potassio-tartrate of iron.* Further, the statement is attributed to me, that usually twenty-four hours of the iron treatment will suffice. Frequently, not usually, was my expression; and I added, "My belief is that in from twenty-four to forty-eight hours the system becomes saturated with it, and that smaller doses are then requisite, or that it may be suspended and chlorate of potash and muriatic acid substituted." I have thought these errors of sufficient importance to induce me to ask the favor of their correction.

Yours truly,

H. GIBBONS.

